# Li Fraumeni syndrome, cancer and senescence: a new hypothesis

**DOI:** 10.1186/1475-2867-13-35

**Published:** 2013-04-15

**Authors:** Pan Pantziarka

**Affiliations:** 1 The George Pantziarka TP53 Trust, London, UK

**Keywords:** Li fraumeni syndrome, TP53, Two compartment tumour metabolism, Reverse warburg effect, Senescence, Autophagy

## Abstract

Li Fraumeni Syndrome (LFS) is a rare autosomal dominant hereditary cancer syndrome characterized by germline mutations in the *TP53* tumour suppressor gene. Sufferers are prone to early onset cancers, particularly sarcomas, adrenocortical carcinoma and breast cancer. Cells from LFS sufferers are known to exhibit telomere dysfunction, genomic instability and spontaneous immortalisation. It is hypothesized that these facets of the LFS host are evidence that the host environment is “primed” for carcinogenesis over and above the lack of p53 tumour suppressor function. Further, it is hypothesized that the host presents an ideal environment for "two compartment tumour metabolism" to take place. Evidence from recent studies supports this new view of LFS and suggests that disrupting certain features of the host environment may markedly reduce the incidence of cancer in LFS sufferers.

## Background

Li Fraumeni Syndrome (LFS) is a rare autosomal dominant hereditary cancer syndrome characterized by germline mutations in the *TP53* tumour suppressor gene. The syndrome is associated with a range of cancers, particularly sarcomas, gliomas, adrenocortical and breast carcinomas as well as other malignancies, particularly during childhood and early adulthood [1,2]. Among women with LFS, the most common disease is breast cancer, with a 49% risk of developing the disease by age 60 [3]. Overall the life-time risk of cancer is estimated at 52% by age 40 years and 80% by age 50 years and for women the life-time risk has been estimated at 100% in one study [2,4,5].

The “two compartment tumour metabolism” hypothesis is a new paradigm that describes a metabolic shuttle between autophagic cells in the tumour stroma and tumour cells [6,7]. The hypothesis suggests that cancer cells induce oxidative stress in the stroma by secreting hydrogen peroxide in surrounding tissues. Cancer-associated fibroblasts respond to this environmental challenge by activation and entry into an autophagic state and undergo mitophagy, mitochondrial dysfunction and a shift of metabolism towards aerobic glycolysis. This metabolic shift in cancer-associated fibroblasts results in the production of high energy by-products such as l-lactate, ketones, glutamine and other mitochondrial fuels that the tumour cells require to drive growth [8,9].

At the heart of this relationship between tumour cells and the surrounding stromal tissues is the autophagic response to oxidative stress [10]. Recent evidence points to a relationship between cellular senescence and autophagy, suggesting that they are part of the same “autophagy-senescence transition (AST)”, and that they both promote the anabolic growth of cancer cells. It also links aging and cancer in a radically new way, suggesting that cancer is a disease of “accelerated host aging” in the tumour stroma [6,11].

## Presentation of the hypothesis

In this paper we present the novel hypothesis that many of the features of the "two compartment" model of cancer, including accelerated host aging, are present in the non-cancerous LFS host, and that people with LFS are therefore "primed" for cancer development over and above a simple loss of p53 tumour suppressor function. Evidence for the hypothesis is reviewed, and the clinical implications discussed.

## Telomeres, senescence and immortalisation

Telomeres are regions of nucleotide sequences that cap the ends of each chromosome and serve to protect the chromosome from recombination or degradation. Successive cell division leads to a shortening of telomere lengths, a process that can lead to chromosomal instability and which is associated both with aging and the pathology of a number of diseases, including cancer. In many respects telomere length can be seen as an indicator of *biological* aging independent of chronological age [12-14]. Crucially, shortened telomeres activate p53 to trigger a DNA damage response that can lead to senescence or apoptosis [15].

Clinical evidence exists that LFS patients have shorter telomeres than age-matched non-LFS individuals [16]. In other studies, children with LFS were shown to have mean telomere length shorter than unaffected parents or siblings [17]. These findings are in line with evidence from LFS fibroblast cell lines derived from patients and in p53−/− and p53+/− knockout mice [18]. In an analysis performed in 2007, shorter telomere length was associated with a younger age of cancer onset in LFS patients, and there was convincing evidence of increased telomere attrition in succeeding generations [19].

Analysis of non-malignant fibroblasts and other cells derived from LFS patients has shown that they display unusual patterns of senescence and that some of them are able to undergo spontaneous immortalisation in vitro. Where control fibroblasts from skin biopsies undergo senescence in the normal way in cultures, some of the fibroblasts from a number of LFS patients enter a long period of growth slowdown and replicative senescence during which they alter morphology, suffer chromosomal damage, including aneuploidy and telomeric association, followed by escape from senescence and the resumption of cell division and replication.

It should be noted that spontaneous immortalisation of human fibroblasts almost never occurs in cultures from non-LFS patients [20,21]. Furthermore, immortalized LFS fibroblasts are not directly tumorigenic when transplanted into nude mice. So while this immortalisation of cells is a necessary condition of malignant transformation it is not a sufficient condition [20].

Escape from senescence is often associated with the over-expression of telomerase reverse transcriptase (TERT), the enzyme complex that re-synthesizes the telomere ‘caps’ at the end of the chromosome. Increased expression of telomerase is common to many cancers, conversely this enzyme is absent from non-transformed cells [22]. Some tissues may be more susceptible to this process than others, as shown in the work of Shay et al. who demonstrated that breast epithelial cells immortalised more frequently than fibroblasts in cultures from an LFS patient [23]. Comparison of the telomerase staining for the different cell types showed significantly higher levels in the breast epithelial cells than in the stromal fibroblasts.

This cell-type specific difference is important in that it is a possible factor in the patterns of cancer incidence in LFS. Breast cancer in LFS affected women is the most common form of the disease, occurring in about 50% of female TP53 mutation carriers (although risk to those that survive to 50 years may be in excess of 70%). Site specific escape from senescence might also explain the prevalence of bone and soft tissue sarcomas, adrenocortical carcinoma and other forms of cancer that are rare in the general population but common in LFS.

**Figure 1:**
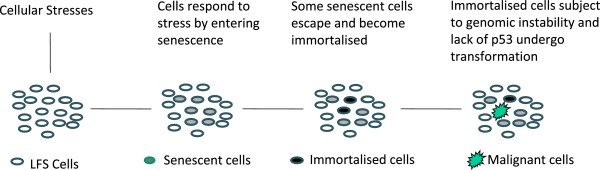
LFS cells with shorter telomere lengths and lacking functional p53 respond to cellular stresses, including oxidative stress, by moving into premature senescence, immortalisation and malignant transformation.

This differential rate of immortalisation and senescence also leads to a situation where small populations of immortalized epithelial cells, subject to mutation and chromosomal change, are surrounded by populations of cells with reduced telomere lengths and already in senescence or becoming senescent in response to oxidative stress (Figure 1). In the context of LFS and the “two compartment” model the stage is set for these immortalized epithelial cells to undergo malignant transformation and to activate the fibroblastic cells in the stroma [24].

### *TP53,* Autophagy and oxidative stress

p53 also plays a role in cellular homeostasis, metabolism and in how cells respond to nutrient deficiency, hypoxia and other stresses. p53 can up regulate oxidative phosphorylation (OXPHOS) by inducing the synthesis of cytochrome c oxidase (SCO2) and down-regulate glycolysis through activation of *TP53*-induced glycolysis regulator (TIGAR) [25-27].

Activated cancer associated fibroblasts undergo transition to an autophagic state according to the “two compartment” hypothesis. Mechanistically, one of the drivers of this transition is an increased rate of oxidative stress due to secreted reactive oxygen species from adjacent cancer cells [28]. There is increasing evidence that p53 is an important regulator of the shift to autophagy. The picture is complex and it appears that p53 may have a dual effect on autophagy, acting as a promoter or inhibitor depending on its localization in the cell [29,30]. However, Tasdemir et al. have shown that inhibition, knockout or knock-down of p53 acts as a potent inducer of autophagy in a range of cell types, including fibroblasts [31]. Lisanti and colleagues specifically link this increased oxidative stress with accelerated host aging in the tumour microenvironment [11,28].

Analysis of the redox parameters in blood samples of healthy LFS patients compared to non-carriers of TP53 mutations found that the LFS mutation carriers had significantly increased indicators of oxidative stress, including a four-fold increase in plasma malondialdehyde levels, indicating increased lipid peroxidation [32]. This is, therefore, further evidence of the priming of the LFS host with the pre-conditions for cancer initiation and progression according to the “two compartment” theory.

### The role of Cav-1

Caveolin-1 (cav-1) is the principal structural component of caveolae, plasma membrane invaginations that participate in diverse cellular activities and are abundant in many cell types. Cav-1 regulates critical cell functions including proliferation, apoptosis, cell differentiation, and transcytosis via diverse signalling pathways. Cav-1 knock-out (KO) mice are an established animal model of premature aging, exhibiting shorter life-span, increased glucose tolerance, insulin resistance and other age-related conditions. Additionally cav-1 KO mice show increased oxidative stress and mitochondrial dysfunction, which are also markers of accelerated host aging [33].

A key hallmark of Lisanti’s “two compartment” theory is a loss of stromal cav-1 and a corresponding up-regulation of cav-1 in tumour cells (i.e. the effects of cav-1 are compartment-specific) [34]. Loss of stromal cav-1 expression is a key indicator of the effect of immortalized epithelial cells on adjacent fibroblasts [35,36].

A recent study by Sherif and Sultan analysed cav-1 expression in non-cancerous LFS fibroblasts and reported that affected family members showed an 88% down-regulation of cav-1 compared to non-affected family members [37]. This new finding is directly as predicted by our hypothesis and confirms that another key feature of the “two compartment” model, (and one that is also related to characteristics of accelerated host aging), is present in the non-malignant LFS host environment.

## Summary

We have outlined a number of important characteristics of the “two compartment” model and have shown how these exist in the non-cancerous LFS host, as shown in Table 1. Furthermore, we have presented evidence that these characteristics are closely associated with the accelerated host aging described in the “two compartment” model of cancer.

**Table 1:** Characteristics of accelerated host aging in tumours and in the non-cancerous LFS host

**Characteristic**	**Two compartment tumour model**	**Non-cancerous LFS host**
Senescent Fibroblasts	✓	✓
High Oxidative Stress	✓	✓
Loss of Stromal Cav-1	✓	✓
Tumour Cells Resistant to Autophagy	✓	✓

### Testing the hypothesis

Our hypothesis predicts that disrupting the “priming” of the host environment will reduce cancer initiation and progression in LFS sufferers and can be tested by undertaking specific interventions. Specifically, there are three possible targets:

• inhibiting senescence in stromal cells

• inducing autophagy in malignant cells/inhibiting autophagy in stromal cells

• interrupting the metabolic shuttle between stromal fibroblasts and tumour cells

These three targets alter the host environment in such a way as to disrupt the “priming” towards the two compartment model, and in theory would markedly reduce the risk of developing cancer in LFS sufferers. A number of important results already suggest that these mechanisms can make a significant impact on cancer risk for LFS sufferers.

Komarova and colleagues showed that the mTOR inhibitor rapamycin, which is known to inhibit cellular senescence, increased lifespan and decreased the incidence of spontaneous tumours in p53 +/− mice [38]. The effect was stronger when started early in life, suggestive of a systemic effect in the host rather than in direct anti-tumour activity. According to our hypothesis rapamycin will decrease the number of cells which enter senescence and hence reduce the number which escape from this state and achieve immortalisation, in turn reducing the number of cells liable to undergo malignant transformation. In other words, decreasing senescence will interrupt the progression shown in Figure 1. Similarly, inhibiting senescence will also stop stromal cells responding to the oxidative stress produced by established tumours by moving into an autophagic state wherein they can “feed” high-energy food to the coupled tumour cells. Thus, retarding the onset of senescence has compartment-specific effects which interrupt the metabolic shuttle between tumour and stroma.

While inhibiting senescence is one possible means of interrupting this metabolic shuttle, another possible mechanism is to induce autophagy in the tumour compartment. The “two compartment” model shows that activated stromal fibroblasts respond to the oxidative stress generated by tumours by becoming autophagic and switching metabolism to aerobic glycolysis, which generates high-energy fuels that can drive the growth of tumour cells [6,8]. However, inducing autophagy in the tumour compartment disrupts the metabolic shuttle as the tumour cells, depending on mitochondrial oxidative phosphorylation, are no longer able to feed on the supply of nutrients from the stroma. Alternatively, inhibiting autophagy in the stroma will deliver the same outcome. One possible mechanism to achieve this is through the use of the autophagy-inhibitor chloroquine, which is currently being used in a number of clinical trials in combination with a range of chemotherapeutic agents.

Finally, the hypothesis suggests that carcinogenesis and disease progression in LFS is sensitive to the metabolic state of cancer associated fibroblasts and adjacent tumour cells. Mutant p53 is stabilised by increased glucose supply, and high levels of mutant p53 act as an inhibitor of autophagy in cancer cells [39]. Reducing glucose supply, therefore, will reduce the stabilisation of mutant p53. It will also decrease the nutrient supply to the stromal fibroblasts which in turn reduces the supply of fuels to “feed” the tumour cells.

Another prediction of the hypothesis is, therefore, that cancer incidence in LFS can be reduced by restriction of the supply of glucose. Options for altering the availability of glucose include dietetic alterations or pharmacological interventions. Supporting evidence for a metabolic influence in LFS carcinogenesis is provided by work on p53 +/− mice, which showed that calorie restriction in adult animals delayed the development of cancer [40].

Chief among the pharmacological interventions is the use of the anti-diabetic drug metformin, already in clinical trials in combination with standard chemotherapy agents. Metformin is known to target many of the pathways affected by dietary caloric restriction, including AMPK, mTOR and IGFR [41]. In the context of the LFS phenotype it would have dual effects. First it acts to restrict the supply of glucose, through the inhibition of hepatic glucose production, to activated stromal cells. Secondly it can act to block mitochondrial oxidative phosphorylation in the tumour cells, hence acting to starve cancer cells through two distinct pathways. There is also some evidence that metformin can selectively induce apoptosis in p53-deficient cells under-going nutrient stress, which is of significant interest [42].

## Implications of the hypothesis

In LFS cancer initiation is more likely than in the general population and may be triggered by the genetic instability that results from shortened telomere lengths, which may be exacerbated by high basal levels of oxidative stress, and by a lack of functional p53. Once cancer is initiated the host environment is already in a state where stromal fibroblasts respond to tumour cells by becoming activated and moving into a state of autophagy, mitophagy and switching metabolism to aerobic glycolysis, thereby feeding the tumour cells with the high energy by-products of this form of metabolism. In short, cancer in LFS patients rapidly moves to a state of “two compartment” tumour metabolism.

To date the common understanding has been that LFS patients are at greater risk of developing malignancies because of the accumulation of secondary mutations over and above the mutated *TP53*. However, the "two compartment tumour metabolism" hypothesis and the additional data outlined above suggest that p53 loss in the stroma accelerates the process of their recruitment by immortalized epithelial cells to promote tumour formation. Thus malignancy in LFS is associated with cellular senescence in stromal cells in response to increased oxidative stress from epithelial cells, a process which may be regarded as a form of accelerated host aging [11].

The hypothesis outlined here is consistent with the patterns of cancer incidence in LFS affected families, for example, the tendency to early onset cancers of specific tissues, such as breast cancer or soft tissue or bone sarcomas, rather than an across the board tendency to all forms of cancer. In particular, it may shed light on the paradox that LFS patients do not show an increased incidence of cancers related to tobacco, environmental toxins or occupation, as would be expected if damaged DNA repair mechanisms were the primary outcome from loss of *TP53* function [43]. It may also explain the higher incidence of cancer before the age of 40, as those individuals with the “more aged” LFS phenotype (shorter telomeres, fibroblastic cells more likely to move into senescence and autophagy, higher levels of basal oxidative stress, greater loss of cav-1) develop cancer at a younger age than those with a more normal telomeric profile (a “less aged” phenotype).

This shift in emphasis from a focus on accumulated DNA damage to cell-type interactions and accelerated aging has important therapeutic implications for LFS. Currently there are no cancer prevention strategies in place for LFS sufferers. Newly diagnosed patients are subject to varying levels of surveillance but are offered no practical steps to reduce the risk of developing malignancies other than bilateral risk reducing mastectomy in women. This imposes high levels of stress on patients and their families, particularly for parents of LFS children and in families with extensive cancer histories.

The hypothesis outlined here opens the door to active chemo-preventative strategies in terms of autophagy inhibition, steps to reduce oxidative stress and so on. Drugs such as the anti-diabetic drug metformin, the autophagy inhibitor chloroquine and other agents with low toxicity, including anti-oxidants, may also be worthy of further investigation in LFS families.

Research is also warranted to ascertain whether shorter telomere length, reduced cav-1 expression or increased oxidative stress have prognostic significance in LFS.

## Competing interests

The author declares that he has no competing interests.
